# Systematic Parameterization, Storage, and Representation of Volumetric DICOM Data

**DOI:** 10.1007/s40846-015-0097-5

**Published:** 2015-11-18

**Authors:** Felix Fischer, M. Alper Selver, Sinem Gezer, Oğuz Dicle, Walter Hillen

**Affiliations:** FH-Aachen, Juelich Division, Medical Informatics Laboratory, Aachen, Germany; Nautavis GmbH, Linnich, Germany; Electrical & Electronics Engineering Department, Dokuz Eylul University, 35160 Izmir, Turkey; School of Medicine, Radiology Department, Dokuz Eylul University, Izmir, Turkey

**Keywords:** DICOM, Grayscale Softcopy Presentation State (GSPS), Compression, Visualization

## Abstract

Tomographic medical imaging systems produce hundreds to thousands of slices, enabling three-dimensional (3D) analysis. Radiologists process these images through various tools and techniques in order to generate 3D renderings for various applications, such as surgical planning, medical education, and volumetric measurements. To save and store these visualizations, current systems use snapshots or video exporting, which prevents further optimizations and requires the storage of significant additional data. The Grayscale Softcopy Presentation State extension of the Digital Imaging and Communications in Medicine (DICOM) standard resolves this issue for two-dimensional (2D) data by introducing an extensive set of parameters, namely 2D Presentation States (2DPR), that describe how an image should be displayed. 2DPR allows storing these parameters instead of storing parameter applied images, which cause unnecessary duplication of the image data. Since there is currently no corresponding extension for 3D data, in this study, a DICOM-compliant object called 3D presentation states (3DPR) is proposed for the parameterization and storage of 3D medical volumes. To accomplish this, the 3D medical visualization process is divided into four tasks, namely pre-processing, segmentation, post-processing, and rendering. The important parameters of each task are determined. Special focus is given to the compression of segmented data, parameterization of the rendering process, and DICOM-compliant implementation of the 3DPR object. The use of 3DPR was tested in a radiology department on three clinical cases, which require multiple segmentations and visualizations during the workflow of radiologists. The results show that 3DPR can effectively simplify the workload of physicians by directly regenerating 3D renderings without repeating intermediate tasks, increase efficiency by preserving all user interactions, and provide efficient storage as well as transfer of visualized data.

**Electronic supplementary material:**

The online version of this article (doi:10.1007/s40846-015-0097-5) contains supplementary material, which is available to authorized users.

## Introduction

Digital Imaging and Communications in Medicine (DICOM) [1] is a standard for the handling, storage, and transmission of digital medical images and related information. It is the most universal standard in digital medicine and its widespread acceptance together with developments in computer technology has enabled many improvements in the way hospitals view their images. Currently, tomographic modalities [e.g., computed tomography (CT), magnetic resonance imaging (MRI)] produce hundreds to thousands of two-dimensional (2D) cross-sectional images (slices) in DICOM format for each scan.

During a diagnosis, several image processing steps are utilized. These steps determine the visual appearance of the image on the monitor. However, the DICOM standard only defines the archiving of the original image data in Picture Archiving and Communication Systems (PACS), but not the settings and annotations made by the radiologists during their diagnosis. Therefore, with each new request to the archiving system, the display parameters must be manually reset. To overcome this, the Grayscale Softcopy Presentation State (GSPS) Storage Supplement [2] creates a DICOM Information Object Definition (IOD) called GSPS, in addition to the definition of DICOM’s grayscale image model. This object contains an extensive set of parameters, namely Presentation States (PR), defining how a particular image or set of images should be presented to the user.

The Two Dimensional Presentation States (2DPR) extension of the DICOM standard allows all the parameters that influence the presentation (e.g., shutters, annotations, contrast, and brightness) to be saved using a parameterized system. A 2DPR object does not generate a copy of the image data; instead, it includes only references to the original data and thus has a small file size. This allows easy transfer to existing PACS and prevents unnecessary duplication of image data. 2DPR can also be used for efficient image distribution between clinical departments to ensure that images can be viewed by physicians in other disciplines with the optimal settings. Approaches for parameterizing the representation of medical data have also been applied to radiotherapy [3], security [4], and other extensions [5].

Three-dimensional (3D) medical imaging usually requires more processing before visualization compared to 2D imaging. In the workflow of a radiology department, a physician, who uses a clinical 3D application, uses several pre-/post-processing, segmentation, and rendering techniques to generate the final 3D rendering. As volume visualization and analysis become a key tool in a variety of health care applications (e.g., radiation oncology, surgical planning, and education), the use of these techniques has become more important [6, 7].

Once the final 3D rendering is obtained, current methods use video/image exporting to save the rendering result. The options for storing the 3D representation of the segmented volume data are limited to images (e.g., JPEG or TIFF), animations (e.g., MPEG or AVI), or 3D movies with pseudo-interaction (e.g., QuickTime, Virtual Reality object movies). Interactions with the objects are available to a very limited extent, if possible at all. Subsequent adjustments to the representation or continuous optimization of the steps of the visualization process are not possible. Thus, it is not possible to return to a volume at a later time to make small modifications (e.g., slight adjustments in segmentation parameters, lighting/shading models, and color/opacity values) to obtain a new rendering result. Moreover, exported videos are usually very large in size in order to include all information at the necessary quality. This requires a significant amount of storage space and introduces challenges in data transfer.

An early approach [8] for the structured storage of visualized volume data defines certain elements, such as transformation and color assignments, and applies them to the original volume data. The data are stored in an XML-based format, but several parameters are not included in the parameterization. There are ongoing attempts for N-dimensional (ND) PR, which is being planned by the DICOM committee (i.e., Working Group-WG-11) to describe the display presentation state for service-object pair (SOP) instances for multi-dimensional PR. One of the most recent updates was on Planar MPR Volumetric Presentation State [9], which is planned to be followed by curved MPR, maximum intensity projection (MIP), and volume rendering PRs. WG-11 announced various use cases of ND PRs such as 3D acquisition modalities and post-processing systems, and tailored viewing protocols [e.g., surface, volume, static, pre-calculated (cine, fly-through), and interactive]. The requirements (i.e., parameters) for these use cases include, but are not limited to, spatial and threshold masking operations, multiple windows with orthogonal, oblique, or curved display mappings, dynamic behavior for cine, sweep/scroll, and state paths for virtual endoscopy and moving cut planes, linked window state for multi-window display such as MPR, high-level rendering descriptions such as shaders, opacity, and color assignments per voxel, and volume cutting planes. Moreover, there are also ongoing attempts for developing PR representations of voxel-[10] and surface-based [11] segmentations.

**Fig. 1:**
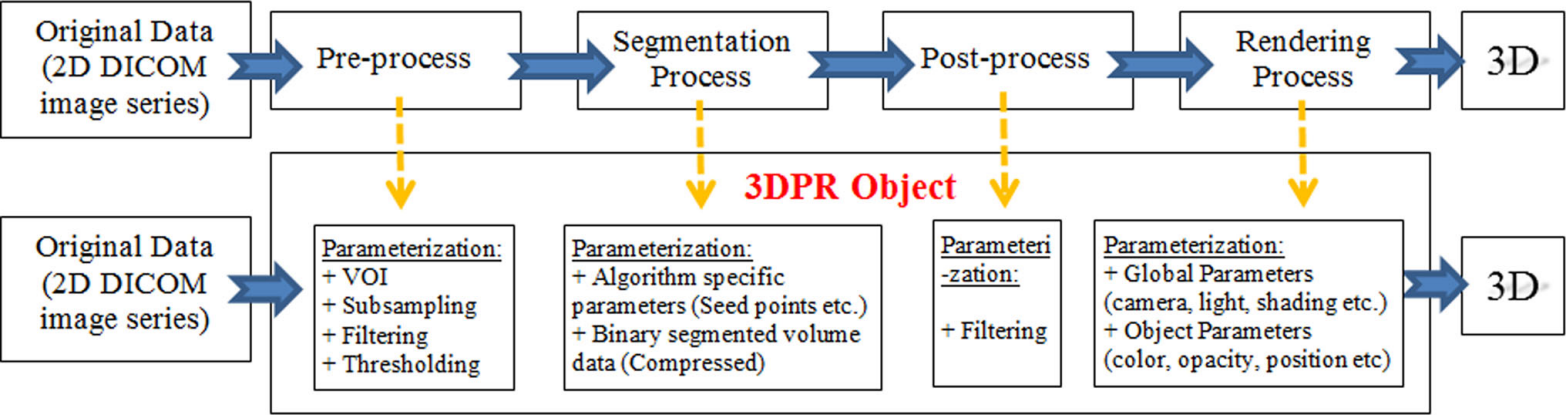
Steps of 3D visualization process (*top*). Repeated operation of process based on previously created parameter set (3DPR) (*bottom*)

In the present study, a data object called 3D presentation states (3DPR) is proposed for storing all parameters and relevant information of 3D visualization. The main idea behind 3DPR is as follows. As 2DPR allows the storage and distribution of the presentation of an image, it can be applied to volume data via 3DPR. Thus, the aim of this study is to develop a systematic and DICOM-conformant parameterization of 3D visualization. This corresponds to parameterizing all procedures of 3D medical visualization (Fig. 1, top row), and storing all necessary parameters and data in a 3DPR object. Then, the 3DPR object can be used to re-run all the procedures automatically to regenerate the 3D visualization (Fig. 1, bottom row). The procedures to be parameterized are pre-processing, segmentation, post-processing, and rendering. The first three procedures are analyzed in another study [12] and shortly reviewed in Sect. 2.1. Instead of storing the segmentation parameters, segmented voxel data can be stored using lossless compression [13]. Using diverse test cases, various compression methods are evaluated in Sect. 2.2. The parameterization of the rendering process is discussed in Sect. 2.3 and the implementation of the system using object-oriented programming concepts is given in Sect. 2.4. The data structure is handled by designing a DICOM-conformant 3DPR object. In Sect. 3, the developed design is applied to three challenging cases. Section 4 gives the conclusions.

## Materials and Methods

This section presents the parameterization of the tasks in Fig. 1 and the compression of the segmented data for efficient storage and use of the 3DPR object.

### Parameterization of Pre-processing, Segmentation, and Post-Processing

In theory, 3D medical visualization can be performed using only the rendering process. However, this has very limited use in clinical practice. To be able to use a visualization for diagnosis, surgery planning, or precise quantitative measurements, the anatomical structure(s) of interest should be segmented [14, 15] using appropriate tools [12, 16–18]. As computer-aided systems evolve rapidly, the need for parameterization of complex methods [19–21] has become more important. Pre-processing of the volume data aims to improve the segmentation process (i.e., data reduction, noise suppression). In the subsequent post-processing, segmentation results are further processed for pruning. Methods for these three procedures, their parameters, graphical user interface (GUI) design for control, and evaluation can be found elsewhere [12, 22]. Some of the parameters are summarized in Appendix 1.

### Compression of Segmented Volume Data

Instead of recalling segmentation parameters and repeating the segmentation process, 3DPR can be used directly for rendering a previously segmented volume. A binarized form of a segmented volume can be defined as a set of voxels, whose values are 1 if they belong to the object and 0 otherwise. Once the binarized form is obtained, the segmentation result can be restored simply by multiplying the original data with the binary segmented data. This makes the binary data an ideal tool for saving segmentation information efficiently [23]. Since the size of the segmented data can be very large, they must be compressed using a lossless technique [24].

To construct adequate lossless data, the segmented data are stored slice by slice in a series of 2D images in uncompressed bitmap (BMP) format. The BMP images are then converted to portable bitmap (PBM) images. In PBM format, the pixels are stored bitwise (i.e., not compressed); thus, the original data size is not reduced. The compression methods used in the present study are as follows: run-length encoding (RLE) is a well-known procedure in data compression that is particularly effective when a symbol repeatedly occurs in the data stream [24]. It is performed with the freely available Java software Birle [25]. The CCITT T.6 standard consists of a combination of binary RLE and modified Huffman coding, where white pixels are corrected using a modified read process [26]. JBIG is a standard specially designed for binary images from the Joint Bi-level Image Processing Group. JBIG2 is the current version of the standard, which was first published in 1999 [27]. The study group behind JBIG was established by the ISO [28] and is also responsible for JPEG2000 [29, 30], which is used for the compression of various types of images based on the wavelet transform [24, 31]. ZIP compression is based on the Deflate algorithm [24], which is composed of two encoding methods: namely LZ77 and Huffman. With the LZ77 method, identical symbol sequences are determined in the data and coded. Then, the symbol sequences are compressed using the Huffman method. The LZ77 method [24] uses a sliding window over the data set. The window consists of two buffers: the search buffer contains the last coded symbols and is used as a dictionary for symbols from the preview buffer. The LZW method [23, 24] uses separate windows instead of sliding ones. During the encoding process, the dictionary is continuously replenished and the size of the dictionary is limited by the memory provided. Octree coding is a special case since, unlike the other methods, the segmented data are considered as a volume (i.e., not as a series of slice images). It takes advantage of the spatial structure of the data. Since no software can be found to apply octree, it is implemented and then optimized in this study [32].

**Fig. 2:**
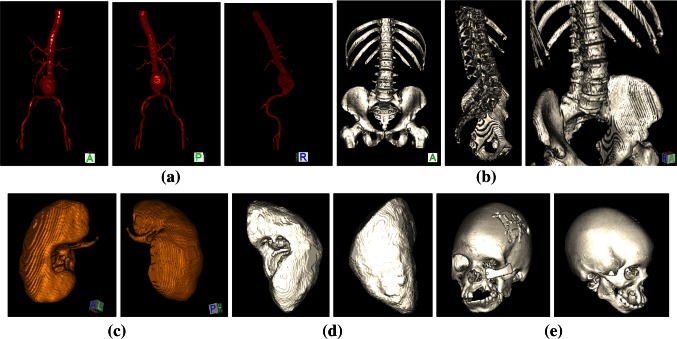
Volume rendered illustrations of segmented medical data sets for testing compression algorithms with **a** aorta, **b** skeleton, **c** MR kidney, **d** CT kidney, and **e** skull

The data sets for testing compression methods were selected from various modalities and anatomical objects with diverse spatial structures so that the performance of the compression techniques can be evaluated for a wide range of applications. 10 data sets are used for each anatomical structure. The “Aorta” data sets were acquired using CT with a contrast medium. They have 288 slices on average with a slice thickness (ST) of 1.5 mm. They have a segmented volume of interest (VOI) of 139 × 322 × 288 voxels, which gives them the largest average dimensions and file size (1631 kB) (Fig. 2a). The “Kidney MR” data sets are coronal MRI image series with 72 slices and an ST of 1.4 mm (Fig. 2c). Their file size is 167 kB. Since the segmentation performance from MRI is limited due to noise, a second type of data set with smoothed versions of the “Kidney MR” series was also used (i.e., Kidney MR-2). The selected VOI has 121 × 52 × 205 voxels. The file size is also 167 kB. The CT kidney data sets consist of 238 slices with an ST of 1 mm and a VOI of 114 × 101 × 112 voxels (Fig. 2d). The file size is 166 kB. The skull CT data sets consist of 361 slices with an ST of 0.7 mm and a VOI of 175 × 214 × 302 voxels (Fig. 2e). The file size is 1389 kB. Finally, “Skeleton” includes ribs and hips from CT (288 slices, ST: 1.5 mm). Its VOI is 239 × 146 × 288 voxels and file size is 1232 kB (Fig. 2b).

### Parameterization of Rendering Process

After parameterization of the segmentation or compressed storage of the segmented data, the parameters related to rendering should be stored in order to recall and obtain the exact 3D representation at a later time. Basically, 3D rendering is composed of a camera, light sources, and objects. In VTK [17], these elements are represented by the *vtkCamera*, *vtkLight*, and *vtkProp3D* classes, which are arranged in a pipeline [17]. In this study, the parameterization of the rendering process is divided into two parts: global parameters, which include the properties of the scene (e.g., lights, shading, camera position, projection type), and object parameters, which include the properties of the segmented object (e.g., rendering type, color, opacity).

Global parameters are used to recall a pre-defined scene of a 3D visualization. Many parameters are used, especially for the camera and the light [17]. The first element of the parameterization is the camera. All camera features and functions are implemented in the *vtkCamera* class. The positioning is determined with the *SetPosition*(double, double, double) method, and the orientation is set with the *SetViewUp*(double, double, double) method. The viewpoint is set using the *SetFocalPoint*(double, double, double) method. If clipping planes are defined, their distance from the camera along the projection direction is set using the *SetClippingRange*(double, double) method. Parallel and perspective projections are activated using the *ParallelProjectionOn*()/…*Off*() methods. In the latter case, the opening angle must be indicated with the *SetViewAngle*(double) method. Position and orientation of the camera can be defined interactively. To simplify the computations, camera-related objects are centered at the origin of the coordinate system. The focus is therefore permanently set to (0, 0, 0) and the distances between the clipping planes are fixed at 0.1–1000.0 mm. The *vtkCamera* instance is passed to the renderer with the *SetActiveCamera*(vtkCamera) method, which allows switching among multiple cameras.

The second element of the parameterization is the light, which is represented using the *vtkLight* class. A light source, which is located at infinity, is enabled by default. VTK also supports other types of light via *SetLightTypeToHeadlight*()/…*SceneLight*/…*CameraLight*. Light color and intensity are fixed with the *SetColor*(double, double, double) (i.e., RGB) and *SetIntensity*(double) methods, respectively. The input range is 0.0–1.0 for each input variable.

**Fig. 3:**
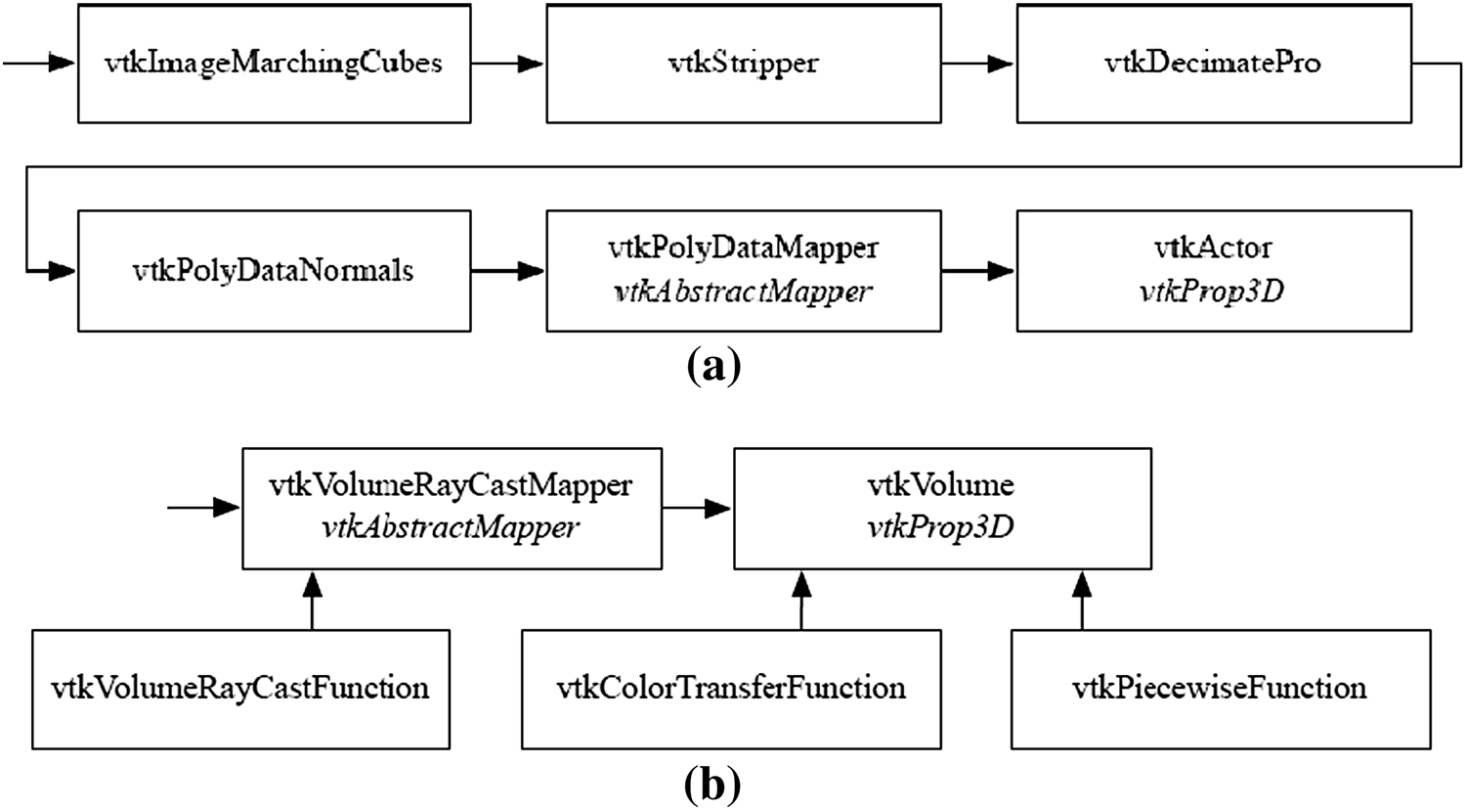
Pipelines of **a** surface rendering and **b** volume rendering. Superclasses are specified in *italics*

Object parameters are related to surface and volume representations. The procedure for the generation and representation of a surface model of the volume data with VTK corresponds to the pipeline [17] (Fig. 3a). The input of the pipeline is the segmented volume data. Based on the segmentation results, the calculation of the mesh of the surface is done using the marching cubes algorithm, which is provided by the *vtkImageMarchingCubes* class of VTK.

The marching cubes algorithm outputs polygons, which are divided into triangles for further processing. In VTK, this is done with the *vtkStripper* class, which requires no parameters. To reduce the memory requirements of the surface and for faster display, the number of triangles is reduced by using *vtkDecimatePro*(). The *PreserveTopologyOn*() method ensures that the topology of the surface is not modified. Since the normals of the surface are required for the lighting model, they can be calculated at this point of the pipeline. The functions for calculating the normals are contained in the *PolyDataNormals* class, which does not require any parameters. The next step is to prepare the triangles so that they can be represented by the graphics card (i.e., transformed into OpenGL). The pipeline element *vtkPolyDataMapper* is used for the conversion of the data. For points that belong to two or more triangles, normals are averaged.

The generated surface model is summarized as an object (i.e., *vtkActor*) that has certain properties (i.e., *vtkProperty*) accessible through the *getProperty*() method. The conditions for the shading options are represented by the methods *SetInterpolationToFlat*(), *…ToGouraud*(), and *…ToPhong*(). *setOpacity*(double) is used to set the opacity (0.0: transparent; 1.0: opaque).

The material and surface parameters are also placed in the *vtkProperty* class. The ambient, diffuse, and specular colors are respectively determined with the methods *SetAmbientColor*, *SetDiffuseColor*, and *setSpecularColor* (each has RGB values of 0.0–1.0). The corresponding weighting factors can be specified using the *SetAmbient*(double), *SetDiffuse*(double), and *SetSpecular*(double) methods, respectively. The specular energy is set using *SetSpecularPower*(double).

The object parameters for volume representations use the visualization pipeline [17] (Fig. 3b). Similar to surface rendering, the segmented data (or original data if there is no segmentation) are the starting point. Ray casting is performed using the *vtkVolumeRayCastMapper* class. Sampling along the viewing ray is done using the *SetSampleDistance*(double) method. The parameterization provides two rendering modes: namely default and MIP, which use *vtkVolumeRayCastCompositeFunction* and *…MIPFunction*, respectively. Both classes are subclasses of *vtkVolumeRayCastFunction*. The mode setting can be changed during a presentation via *SetVolumeRayCastFunction*(vtkVolumeRayCastFunction).

The transfer functions for mapping gray values in terms of color or opacity, and imaging of opacity gradient magnitudes are represented by the *vtkColorTransferFunction* and *vtkPiecewiseFunction* classes. They can be set using the *SetColor*(vtkColorTransferfunction), and *SetScalarOpacity*/*SetGradientOpacity*(vtkPiecewiseFunction) methods. For transfer function specification, an editor was developed according to a previous study [33]. Once the creation of the visualization pipeline is completed, the representation of the solid model is analogous to the surface model, i.e., the instance of *vtkVolume* (subclass of *vtkProp3D*) is passed to the instance of *vtkRenderer* by the method *Addvolume*(vtkProp3D). A list of the above-mentioned object parameters for both volume and surface rendering is given in “Parameterization of Rendering (Object Parameters)” section of Appendix 1.

### Implementation of System

**Fig. 4:**
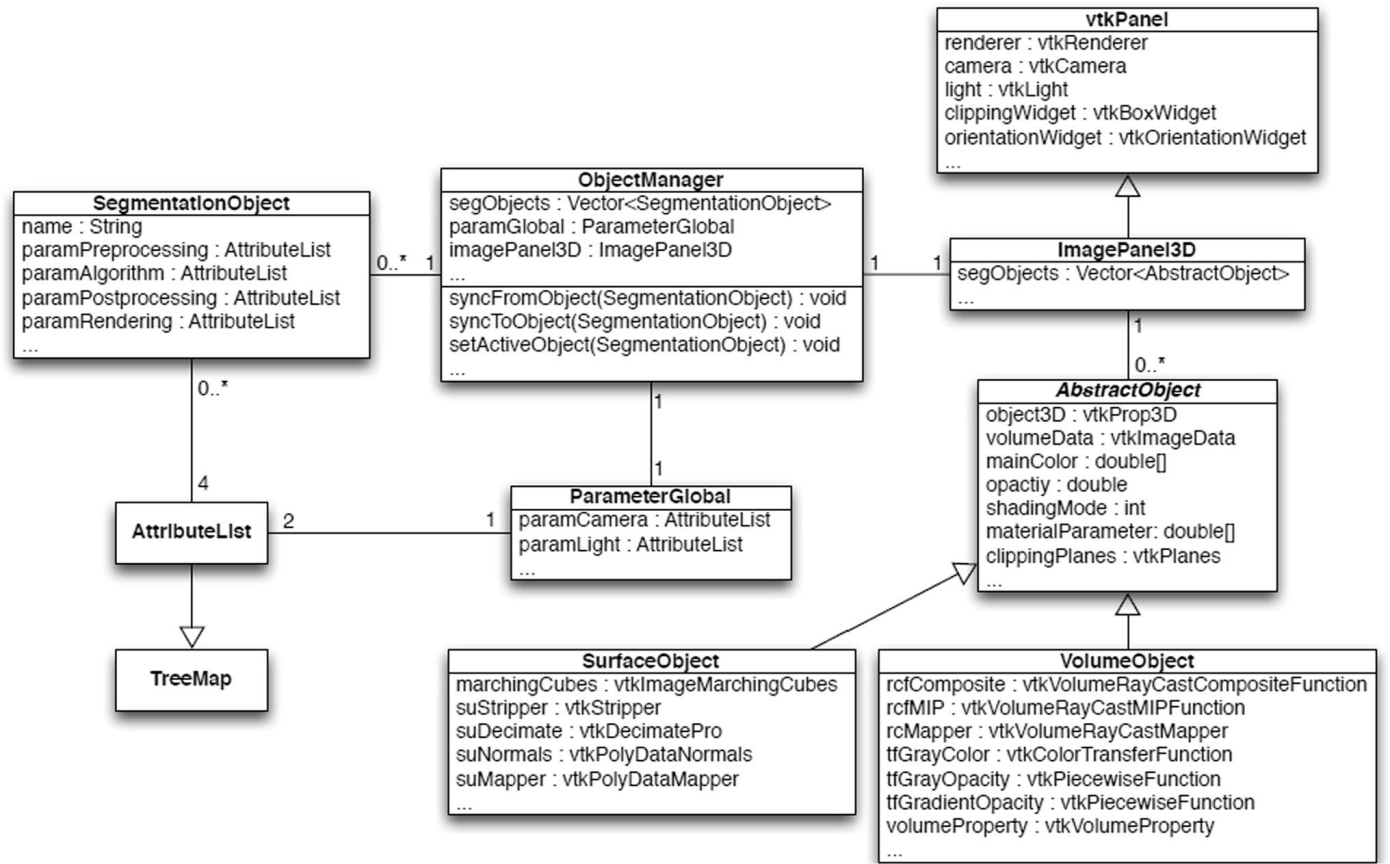
UML class diagram of main system components

The software design and the development of the framework application were done based on object-oriented programming concepts. The software design of the main components is presented in the form of a UML class diagram (Fig. 4). The object manager [12], which controls the file system operations of 3DPR, is represented as an interface class between the parameters and the 3D view.

From pre-processing to rendering, the individual parameters of an object are summarized in the SegmentationObject class, along with the user-assigned object names. According to the processing steps, the parameters are divided into four classes of AttributeList, which is an associative memory structure. The data structure behind AttributeList is a hashtable, in which the elements are stored in a sorted binary tree. These properties are inherited from the class TreeMap from the Java Collections Framework. AttributeList adds additional properties and manages the individual parameters (i.e., attributes) that are already in a DICOM-compliant structure as “DICOM Attributes”. The generation and loading of 3DPR are considerably simplified since this data structure can be transferred directly to medical databases (e.g., PACS).

The GUI for setting the parameters is provided in the ObjectManager class, which has references to all the instances of SegmentationObject and to the ParameterGlobal classes. Synchronization of the parameters is possible in both directions: the parameters of the GUI can be written to the data structures and the GUI can be set based on the data structures. The latter is required for loading a previously saved 3DPR object. When an item (i.e., 3DPR object) is selected in the object manager, the SetActiveObject(SegmentationObject) method is called. First, the parameters of the previously selected object from the GUI are written to the data structure, and then the GUI is populated with the parameters of the selected/loaded 3DPR object.

The 3D visualization part of the software is implemented in the ImagePanel3D class. This class is a subclass of vtkPanel, which belongs to VTK and represents the Java bridge to the vtkRenderWindow class. As shown in the UML class diagram, this class consists of vtkRenderer, vtkCamera, and vtkLight (described in the previous section). vtkBoxWidget and vtkOrientationWidget represent the widgets for adjusting the clipping planes and representing the orientation cube, respectively. For each 3DPR object, an instance of a subclass of the abstract object is kept ready in the class ImagePanel3D. The storage of all parameters is done with a DICOM information object, which is based on the structure of 2DPR, but extended with additional modules.

#### DICOM Information Objects and 2DPR

Information objects are defined in part 3 of the DICOM standard [1]. An information object comprises a series of associated information entities. One unit of information is associated with one or more modules, in which attributes are stored. In the DICOM standard, two types of IOD exist: normalized IOD and composite IOD.

**Fig. 5:**
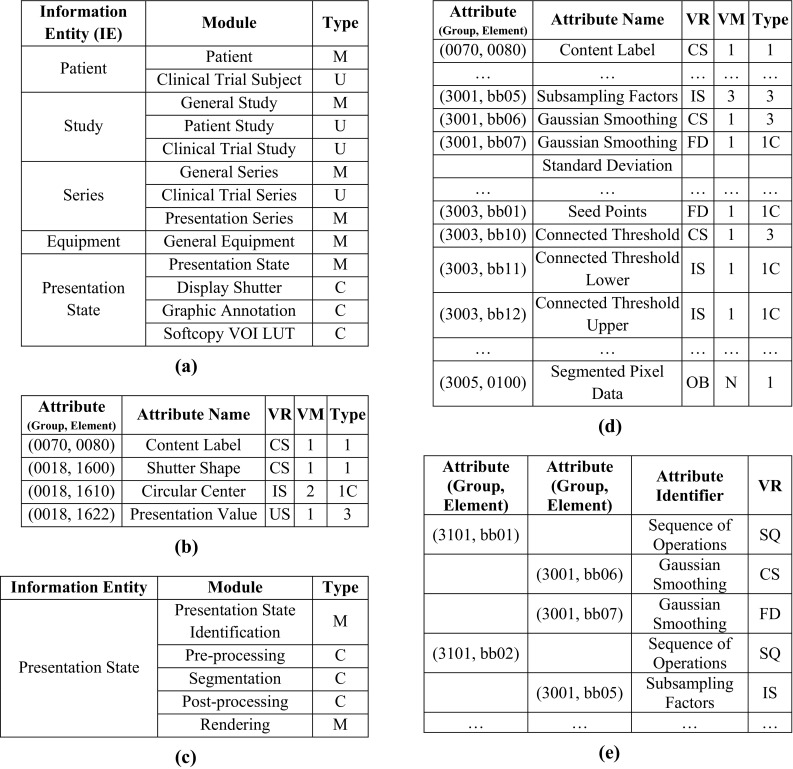
**a** 2DPR IOD modules (excerpt). Complete overview of all modules of PR information object can be found elsewhere [4, 5]. **b** Excerpt from DICOM Data Dictionary. *VR* stands for “Value Representation” (data type), and *VM* stands for “Value Multiplicity” (frequency). **c** 3DPR IOD modules (extract). **d**, **e** Excerpts from the Data Dictionary for the 3DPR. “FD” and “OB” means “Floating Double” and “Other Byte”, respectively

Each 2DPR corresponds to a DICOM information object in the construction of a composite IOD. The attributes of the individual modules define which operations are to be applied to a particular image (or series of images). There are 27 different modules for 2DPR, which are divided into five different units (Fig. 5a). There are modules that must be available in an information entity (M = Mandatory), modules that exist under certain conditions (C = Conditional), and modules that are optional (U = User Option). Patient information entities (such as Study, Series, and Equipment) are not discussed here since they include only demographic information about patients.

As shown in Fig. 5a, the Presentation State module is mandatory, as it contains general information about the PR, such as a unique ID, a name, and references to the associated image data. The other modules, including graphical annotations (i.e., Graphic Annotation), shutter (i.e., Display Shutter), and contrast/brightness settings (i.e., Softcopy VOI LUT), are required only if information is included.

Within a module, several attributes are defined to describe an object more accurately. The allowed attributes are defined in the DICOM Data Dictionary part of the standard. Each attribute is given an identifier, which consists of a group and an item number in hexadecimal code, a name, a Value Representation (VR), and a frequency value (Value Multiplicity: VM). The DICOM standard allows third-party manufacturers to assign private attributes.

Figure 5b shows an extract from the DICOM Data Dictionary with attributes from the PR module. A content label must be defined and contain a brief description of the PR. The data type CS means “Code String”. The attribute “Center of Circular Shutter” is the center of a circular form that must be defined only if the attribute has the value “Shutter Shape Circular”. It has the Integer String (IS) data type. “Shutter Presentation Value” (data type: “unsigned short”) is the gray value of the shutters (0000: black; ffff: white).

Different types of attributes can be described as follows. Type 1: Attribute must be defined in the data structure and must have a valid value. Type 2: Attribute must be defined in the data structure, but does need not to contain a value. Type 3: Attribute is optional. Type nC: If another attribute is present in the data structure, attribute must be coded by taking the n-type into account, where n is equal to 1 or 2 (Fig. 5b). Attributes do not only consist of values, but also include a sequence of other attributes. For such cases, the data type is Sequence of Items (SQ) (Fig. 5e).

#### Implementation of 3DPR

As mentioned, 3DPR should be compatible with 2DPR to ensure interoperability with existing DICOM systems. Therefore, 3DPR is based on the structure of 2DPR, and extends it by using various modules. The “Presentation State” module of 3DPR (Fig. 5c) includes general information identical to the corresponding module of 2DPR (Fig. 5a). In accordance with the conditions laid down for parameterization (Sect. 2), all parameters of the individual steps up to the 3D visualization are included by adding pre-/post-processing, segmentation, and rendering to information entities. As shown in Fig. 5c, modules “Presentation State” and “Rendering” are mandatory (i.e., M), while the other three modules must only be present if they are applied (i.e., C). Without the “Presentation State” and “Rendering” module attributes, a meaningful representation is not possible. However, pre-/post-processing may not be used, as well as segmentation. For instance, if only a transfer function is applied to the original volume data or an MIP takes place, only the parameters of the “Rendering” module are needed.

For the attributes in the add-on modules, new group numbers are assigned. All attributes from the pre-/post-processing modules belong to group number 0x3001, while attributes from the segmentation module belong to group 0x3003 and those from the rendering module belong to group 0x3005. The attributes of pre-/post-processing are not distinguished, since their parameters are identical (with the exception of the VOI and subsampling). Figure 5d shows an excerpt from the Data Dictionary for 3DPR. The value of the subsampling attribute consists of three factors (i.e., an integer for each dimension) and is optional. Another option is the Gaussian smoothing attribute. If it exists, the standard deviation must be specified (type 1C) (Fig. 5d).

For each entry in the object manager, the individual parameters of the pre-/post-processing, segmentation, and rendering of the ObjectManager or SegmentationObject class are queried to AttributeList. The same applies to the rendering process parameters, such as the camera and the light source (i.e., global parameters). The AttributeList class manages the parameters in a DICOM-compliant way. An attribute list consists of attributes, their structure (group and element number), and their properties. The segmented volume data (i.e., attribute SegmentedPixelData) are in binary form and encoded in the JBIG2 format (Sect. 2). In 3DPR, they are represented by the attribute (0x3005, 0x0100), which consists of a series of bytes (i.e., raw data of the data stream of JBIG2).

Since the order of operations may affect the result or some operations may be applied repeatedly, such series of attributes are set to 0x3101 with VR = SQ. These operations are executed in the order of their group and element numbers. For example, Fig. 5e shows an application where Gaussian smoothing is applied twice and then subsampling is performed. For the “Rendering” module, no specific definition of the order is necessary and the individual parameters cannot be applied more than once.

As a result, each entry in the object manager stores an “object” and its “scene”. Thus, a 3DPR represents a rendering of entries in the object manager. When more than one entry (i.e., object) is rendered, the global parameters, such as lighting and camera position, determine the “scene”. The object parameters (such as color and rendering type, i.e., volume or surface) are stored in 3DPR together with segmented data of the corresponding object. The benefits and the side effects of storing the segmentation data inside the 3DPR rather than separating it out into its own referenced DICOM instance are discussed for an aneurysm application (Sect. 3.2.3).

#### Validation

The DICOM standard is an extensive and complex standard. Therefore, it is necessary to check the generated 3DPR structure and its syntax in terms of the DICOM conformance to ensure that it can be used properly in all DICOM environments. An overview of different validation tools for this purpose can be found elsewhere [34]. In this study, the command-line-based software DCMCHECK, which supports the examination of individual DICOM objects and is available for various operating systems [35], is chosen. Specifically, the conformity with regard to the IODs (part 3 of the standard), the data structures and encodings (part 6), and the consistency between header and meta-header data (part 10) is checked and validated.

Various 3DPR objects (i.e., test cases) are used during the validation. These objects are created by applying the developed visualization framework to various clinical applications, which are selected to contain a wide range of possible parameter combinations.

## Results

The application and use of 3DPR are evaluated both quantitatively and qualitatively. Section 3.1 introduces the quantitative compression results for segmented data. Section 3.2 presents qualitative discussions on the use of 3DPR in three challenging clinical applications. Section 3.3 presents the quantitative measures for computational performance, usability, and data storage for comparing 3DPR and existing techniques.

### Performance of Compression Methods

Table 1 shows the results in terms of the compression ratio for the anatomical structures presented in Sect. 2.1. The size of the PBM data corresponds to the uncompressed size of the average of the 10 original data sets [(width × height × depth of the VOI) × 1 bit + header of the PBM data]. Thus, the values in Table 1 show how much the PBM is compressed in percentage. By far the best result (91.36 %) is obtained for the aorta. Good compression ratios are also obtained for the skeleton (73.48 %) and the kidney CT (68.63 %). These three test cases consist of large homogeneous regions, which are beneficial for compression methods. Lower compression rates are obtained for the kidney MR-2 and the skull, both of which have fine-grained structures inside the organs. In kidney MRI, the strong smoothing during pre-processing does not lead to substantial improvement in compression. Therefore, the high compression ratio cannot be attributed to a large amount of noise in the original data. Although octree is the only method that takes advantage of correlations in 3D, its compression ratio (31.45 %) is very poor compared to those of the other methods. The compression times for all methods are less than 1 s except that for octree, which required a few seconds.

**Table 1:** Compression ratio in %

	Aorta	Kidney CT	Kidney MR	Kidney MR-2	Skull	Skeleton	Avg.
JBIG2	98.89	96.51	94.73	95.33	95.27	95.95	96.11
CCITT T.6	97.30	93.51	91.16	91.16	93.05	93.85	93.34
ZIP	98.04	92.31	90.56	91.16	90.83	92.07	92.50
LZW	95.90	87.51	86.99	87.58	84.32	88.92	88.54
RLE	95.04	68.93	74.48	73.88	65.33	82.70	76.73
Octree	89.74	40.16	10.13	16.09	−0.29	32.87	31.45
JPEG 2000	84.98	10.79	−11.32	−5.96	−3.80	28.27	17.16
Average	91.36	68.63	62.19	63.75	61.09	73.48	

In order of compression ratio: from highest to lowest

Negative values indicate increase in data size)

The JBIG2 method outperformed the other techniques used in this study and it reduced the data size below 5 % for all anatomical objects. Moreover, the compression ratio of the JBIG2 method reduces the segmented binary volume data to acceptable file sizes for 3DPR. Another important advantage is that the compression time of JBIG2 is negligible (less than 1 s). Therefore, the JBIG2 method can be used for effectively compressing binary segmented volume data.

Although the JBIG2 method shows the best performance, it is actually optimized for the data reduction of binary 2D images. It would be better to use a method that can be applied directly to the volume data without having to split them beforehand into individual slices. In this way, correlations in the data may be used not only in two, but also in three dimensions. This requirement can be achieved by modifying the JBIG2 method as follows. The high compression factor is partly due to the use of a context-based coding, in which the context of the neighbors of the pixel are encoded. In the current version of JBIG2, the neighbors are always in the same plane or slice as the pixel to be encoded. By enlarging the vicinity to adjacent slices, the JBIG2 method can be used without a change in the actual encoding process with a presumably more efficient compression of binary volume data.

Finally, it is important to point out that the compression methods used in this study were selected to determine the best performance for binary volume compression in experiments, not to determine how the segmented data should be stored in a clinical system (which should only be in DICOM format).

### Clinical Utility of 3DPR

The system was evaluated over a period of nine months at the Radiology Department of Dokuz Eylul University (DEU) hospital. Three teams of radiologists used the developed program for the clinical cases of living donated liver transplantation (LDLT), abdominal aortic aneurysm (AAA), and renal tumors. Each group consisted of three radiologists and included at least one resident radiologist and one professor specialized in the corresponding clinical application. These routine clinical cases were selected among many daily tasks in radiology, since they require analysis of segmented 3D data several times by radiologists and other physicians (such as surgeons). The segmentation quality, software usability, and the integration of the entire system in a radiological environment were evaluated using one-to-one studies, quantitative assessments, and surveys.

Software can be evaluated using three methods: analysis during development, comparison of multiple software systems, and sole use of software. The first method is called formative evaluation. The other two methods are similar to each other and they are only applied to developed software systems. Together, they are called summative evaluation. In summative evaluation, the focus is on the assessment of software based on predefined criteria. It is a final evaluation, during which it is examined whether and to what extent the implementation is in compliance with the criteria. Mainly quantitative methods such as questionnaires are used. 3DPR was evaluated using questionnaires and interviews.

#### Applicability

Since the applicability of the system is directly related the parameterization, the following issues are discussed. Is the scope of the parameterization sufficient for the selected clinical cases? Are any important parameters missing? Are the parameters grouped meaningfully?

The feedback from the working groups was evaluated individually for the processing steps: pre-processing, segmentation, post-processing, and rendering. The results of the three groups (LDLT, AAA, and renal tumors) are presented in parallel, unless there is an issue specific to a test case.

During the evaluation phase, the most common tool used was the interactive VOI selection. The current version of the 3DPR provides VOI determination only as a cuboid. All three groups indicated that a more flexible determination of the VOI is needed for complex shaped objects such as the liver or aorta to improve segmentation performance, decrease processing time, and obtain more effective transfer function specification. A flexible VOI requires a more complex scheme for its parameterization and storage, whereas a cuboid can be clearly defined in terms of three variables.

Other pre-processing steps can also be easily encoded into 3DPR and more options can be added where necessary. For instance, between several options for filtering, anisotropic diffusion [16] was chosen as the main method for noise reduction by all groups. The importance of freely selectable filter kernels in clinical practice is low since users lack the required theoretical background to make a choice and the default parameters of anisotropic diffusion mostly provide sufficient performance. The thresholding method for the exclusion of certain Hounsfield or gray values was used only for liver segmentation, where the ribs were removed by thresholding.

#### Pre-evaluation of Living Donated Liver Transplantation

The pre-evaluation of a living liver transplantation donor can roughly be divided into two parts: (1) segmentation of the liver for measuring its volume and (2) analysis of the liver vasculature for surgical planning. Due to their education, radiologists can interpret 3D structures from 2D slices or MIP images. Surgeons prefer 3D rendering, which requires segmentation of the liver by a radiologist in advance. Moreover, necessary 3D rendering parameters should be set for vascular analysis. Currently, in DEU hospital, radiologist-surgeon meetings are based on snapshots and videos prepared by the radiologist.

The 3DPR approach was applied to 21 retrospective cases. It was observed that the application of 3DPR considerably simplifies the workflow and increase the efficiency during this procedure. Since the results of the segmentation of the liver and the parameters for 3D rendering can be saved, the fully parameterized 3D model can be restored immediately at the radiologist-surgeon meetings. Using 3DPR, it is possible to interact with the 3D liver instead of watching screenshots and videos. The same advantage applies for MIP, which can be modified during the meeting based on the expectations of the surgeon.

A further advantage of the concept is the use of 3DPR in the operating room, where any interactive 3D model can be restored instantly during surgery. Considering the critical timings of living liver transplantation, this offers a decisive advantage (i.e., the donor and patient lie adjacent to each other, a resected part of the liver from the donor is transferred directly into the patient; unlike the kidneys, there is no machine to cover liver function for some time). For instance, in case of any complications, other 3DPR objects can be used for guidance by visualizing previously planned alternatives.

#### Analysis of Abdominal Aortic Aneurysms

The analysis of AAA requires a complex workflow, which consists of 3D visualization for determination of the stent (graft) size and location. This is a critical analysis, since misplacement of a stent may block renal vessels departing from the aorta and may cause the kidneys to fail. Moreover, incorrect stent size requires further surgery, increase cost. Another 3D analysis is required after the surgery. The aim of this second analysis is to visualize the stent together with the aorta and compare the final rendering to that obtained before surgery. Currently, both tasks are performed via MIP in DEU hospital. In this study, the 3DPR approach was applied to 12 cases and compared to the existing method. The use of 3DPR allows exact recovery of the segmentation and representation of 3D AAA before the implantation of the stent for comparing the aorta after surgery. Accordingly, pre- and post-surgery 3D data can be compared interactively. If the parameters of the acquisitions allow (i.e., if they are similar enough), the 3DPR prepared for the pre-operative data can be used directly to visualize the post-operative data. Thus, a direct comparison is possible.

The clinical evaluation of AAA revealed another important advantage of the 3DPR object. It is possible to parameterize the complete system with more than one object and control them with the object manager GUI. In AAA, the aorta and surrounding vessels are segmented with the region growing method (parameters: user-inserted seed locations and upper and lower thresholds) and stored as the first 3DPR object. The stent is visualized with several transfer functions (parameters: opacity maps), which are localized at different parts of the vascular tree. Utilization of several transfer functions is necessary for clear rendering due to local variations in the Hounsfield range. The vascular tree and stent objects have their own parameters and can be used individually (i.e., visualization of only aorta or only stent). When they are visualized together, global parameters determine the overall scene.

#### Renal Tumor Diagnostics

In the case of renal tumors, the typical workflow of the radiology department is as follows. First, an analysis of the tumor (i.e., to determine whether it is malignant) and a coarse segmentation is performed (i.e., to determine its approximate size) by the radiologist to decide whether it is necessary to direct the patient to the hospital council. In the council, further discussions are made by physicians from different fields of expertise. Based on the conclusions, the need for a surgical operation is decided. In case of surgery, a finer segmentation is performed in the radiology department. The first (coarse) segmentation is usually performed by a resident, while the second (finer) one is performed together with the responsible senior physician and/or professor. This workflow requires multiple segmentations and visualizations. The senior physician may ask for additional information that requires new segmentation and visualization tasks. In this case, 3DPR can simplify the process as follows: the results prepared by the resident can be parameterized and stored. Based on the feedback of the senior physician, fixes and improvements to the segmentation can be performed immediately, since all segmentation parameters are stored in the 3DPR object.

Furthermore, the 3DPR allows the radiologist to refine the coarse segmentation performed in the first round of the analysis. The coarse segmentation, which does not take much time, can be restored to initialize the second round of finer segmentation and 3D visualization. Since a revision history is not implemented in the current version, 3DPR objects simply store the last set of parameters. Thus, at each round of analysis, the user can overwrite a 3DPR object or create a new one depending on the chosen save option.

### Computational Performance

During the evaluation phase, the time required for generating and displaying a parameter set for a volume data was found acceptable by the users. Depending on the number and size of the segmented objects, the restoration time was less than 2 s (for a PC with a 2-GHz dual core processor and 8 GB of RAM). A typical segmented aorta has 20 kB of data. It takes less than 1 s to compress and store its 3DPR object. The additional stent representation is exclusively achieved by the application of a transfer function, and therefore requires almost no additional time or file size. In the case of the liver, the result is largely composed of inhomogeneous structures. Therefore, the size of the 3DPR object increases up to 250 kB and the storage time becomes slightly more than 2 s (compression requires the most time).

The total storage needs for each case are presented in Table 2. If video exporting is used, each HD video requires around 120, 66, and 47 MB for LDLT, AAA, and renal tumor analysis, respectively. These values increase to 414, 201, and 154 MB for 1080p resolution, respectively. The same information can be stored using 3DPR objects with sizes of 0.45, 0.61, and 0.28 MB, respectively, which reduce the yearly average storage needs by 99.8 %.

**Table 2:** Comparison of computational performance and storage requirements

	Storage needs (MB)			Cases per year	Total storage (per year) Video (1080p)/3DPR (MB)
	Video (HD)	Video (1080p)	3DPR		
Liver transplantation	120	414	0.45	17–24	8694/9.45
Abdominal aortic analysis	66	201	0.61	36–50	8643/26.23
Renal tumor diagnostics	47	154	0.28	90–140	17,710/32.2

Due to the DICOM conformance of the 3DPR object, it can be stored via DICOM services into an existing PACS. The 3DPR object can be recalled from PACS and transferred to any workstation on the network. A particular advantage of this method of selection is that the original image data is always available, because the 3DPR Object is placed parallel to this data in PACS. There is no additional transmission time because the size of a 3DPR object is negligible compared to the original data.

## Discussion and Conclusion

The aim of the presented study was the full parameterization of a 3D visualization process for medical volume data. The parameters are stored as a DICOM-compliant object called 3DPR. To accomplish this, the entire visualization process was first divided into four tasks: pre-processing, segmentation, post-processing, and rendering. For each of these tasks, methods were selected, parameterized, and implemented.

A special feature of the parameterization is the handling of the binary volume data of the segmented object. This large amount of data must be described by a highly compressive, yet lossless, method that takes the binary structure and the spatial relationships of the segmentation results into account. For this purpose, various compression methods were tested on data sets with different characteristics (such as compactness of the segmented object). The best results were obtained using the JBIG2 method, which achieved an average reduction of approximately 3 % of the original size. The compression time is about 2 s for typical data sets.

The experimented compression techniques were applied to only voxel segmentations. A growing number of applications perform surface segmentations and work with the resulting surfaces, in which the segmented shapes are encoded in a polygonal representation of the surface. An analysis of Surface Mesh Module [11], which is used to encode the surface segmentation data, would be an interesting future work in order to extend the existing Segmentation SOP Class to specify a surface derived from any DICOM modality or non-DICOM measurement technique or even designed surfaces. Moreover, the segmentation methods used in this study all produce binary results. In many novel methods, each voxel may instead represent fractional or probabilistic occupancy, which should be handled with different approaches and techniques in order to obtain high compression ratios.

All parameterization procedures are realized in a complete system programmed in Java. For the segmentation and rendering algorithms, the ITK and VTK toolkits are used. A strong emphasis was given to the design of an intuitive user interface during development.

When storing a 3DPR object, all the parameters and data of the 3D visualization process are transferred to a technical form, which expands the existing DICOM Presentation State structure. Any specific parameters for the application are added as “private” (proprietary) DICOM tags. In this way, it is ensured that the parameter set can be integrated into existing image databases (e.g., PACS) without modification. The data size of a 3DPR object depends on the segmented object. For a typical liver segmentation, a 3DPR object is 250 kB. Moreover, preparation and presentation of each 3DPR object takes around 2 s.

The whole system was installed and used over a period of 9 months at the Radiology Department of DEU Hospital. Three teams of radiologists used the system for three clinical cases, which required the segmentation and 3D visualization of the medical data multiple times during diagnosis, planning, and treatment. These test cases included volumetric evaluation and vascular analysis of living liver transplantation donors, 3D measurements for graft selection, surgical planning of AAAs, and volumetric measurements of renal tumors.

The segmentation quality, software usability, and integration of the entire system in a clinic were evaluated. The evaluation shows that the use of 3DPR efficiently simplifies and accelerates the workflow, where repeated applications of 3D visualization and analysis are needed. Moreover, instead of using movies, 3DPR provides great flexibility by allowing direct visualization of segmented data whenever needed. These advantages increase the cooperation of the radiologists with other physicians (such as surgeons), while reducing the workload required for repeated 3D analysis. Full integration of 3DPR objects with PACS via DICOM-compliant data structure enables the use of the developed approach with existing systems.

Overall, the 3DPR approach is found to be effective. The time required for generating and displaying a 3DPR object is acceptable. The evaluation also shows that the workflow for the selected applications through 3DPR can be greatly simplified and accelerated. Moreover, the storage needs for 3D renderings in PACS can be reduced by 99.8 %.

Here, it is important to point out that the main goal of this study was to save and restore 3DPR objects in a standardized way using the developed implementation. However, in order to make the whole system completely interoperable, more design criteria must be met. For example, each certified system has to implement all segmentation and rendering algorithms the same way. This causes a limitation, because ITK and OpenCV differ in their implementations. This is also true in rendering. Even within VTK, the results may differ between different graphics cards (e.g., TextureMapping rendering highly relies on graphics card capabilities).

Another limitation of the approach might be the storage of the segmented volume data within the 3DPR object itself and not referencing it as a separate object. The former is chosen in this study to keep 3DPR objects compact (3DPR is kept as a single file). The latter strategy requires several files, but it is the correct and required way for DICOM conformance.

Future works should focus on the development of an effective 3D compression scheme both for binary and probabilistic segmentation results, improving 3DPR to store the segmented data as a separate object, and extending the design to keep a history of revisions in the 3DPR object.

### Electronic supplementary material

Supplementary material 1 (MP4 19927 kB)

## Appendix 1: Parameter Tables

### Parameterization of Pre-processing

**Table:**

Designation	Parameter	Importance
Volume of interest	$\overline{{O^{\text{VOI}} }}$	Origin in world coordinates
	**r**_*x*_, **r**_*y*_, **r**_*z*_	Length and direction in world coordinates, the three vectors must be orthogonal to each other
	Interpolation	NN (nearest neighbor) or TL (trilinear)
Subsampling	n_x_, n_y_, n_z_	Sampling interval in the x-, y-, z-direction (in the voxel coordinates)
Gaussian filtering	σ	Standard deviation in world coordinates
Anisotrope diffusion	k	Conductance parameter
	t	Time step, the standard deviation of the Gaussian filter $\sigma = \sqrt {2t}$ number of iterations
Gradient magnitude filter		No parameters required
Sigmoid filter	Min, max	Min-/maximum value of the coverage area
	α, β	Define the shape of the mapping curve
Free filter mask	h(m,n,l) k	Coefficients of the filter mask environment, −*k* ≤ *m* ≤ *k*, −*k* ≤ *n* ≤ *k*, −*k* ≤ *l* ≤ *k*
Thresholding	t_lower_, t_upper_, v_outside_	Limits of the gray value interval Gray value outside the interval

### Parameterization of Segmentation

**Table:**

Designation	Parameter	Importance
Connected threshold	S = {s_1_, s_2_, s_3_…} with s_n_ = {s_nx_, s_ny_, s_nz_…}^T^ t_lower_, t_upper_ Limits of the selected gray value interval	Positions of the seed points
Fast marching	S = {s_1_, s_2_, s_3_…} with s_n_ = {s_nx_, s_ny_, s_nz_…}^T^ t_max_ Maximum number of iterations	Positions of the seed points

### Parameterization of Rendering (Global Parameters)

**Table:**

Designation	Parameter	Importance
Camera	C_pos_	Position in world coordinates
	C_up_	Orientation in world coordinates
	C_fp_	Focus (Focal Point) in world coordinates
	d_near_, d_far_	Distance of clipping planes from the camera position
	Projection	PAR (parallel) or PER (perspective)
	α_p_	Opening angle (f perspective projection)
Light source	Position	S_L_ (Scene Light), C_L_ (Camera Light), H_L_ (Head Light)
	L_pos_	Position in world coordinates (not for HL)
	L_fp_	View point in world coordinates (not for HL)
	L_c_	Color (RGB tuple)
	L_i_	Intensity

### Parameterization of Rendering (Object Parameters)

**Table:**

Designation	Parameter	Importance
Clipping	E_p,i_ where i = 1…6	Start point of the level i in world coordinates
	E_N,i_ where i = 1…6	Normal of level i in world coordinates
Representation (surface rendering)	O_c_	Surface color (RGB tuple)
	O_op_	Surface opacity
	Shading type	F (Flat), G (Gouraud), P (Phong)
Representation (volume rendering)	TF_c_(I)	Transfer function (gray value → color value)
	Tf_op_(I)	Transfer function (gray value → opacity)
	Tf_op_^grad^(|∇I|)	Transfer function (gradient → opacity)
	Mode	STD (standard) or MIP (maximum intensity)
	s_xy_	Sampling in 2D
	s_t_	Sampling along the ray in world coordinates
	Interpolation	NN (nearest neighbor) or TL (trilinear)
	Priority	CLA (Classification) or INT (interpolation)
Material/surface	k_a_	Ambient weighting factor
	k_d_	Diffuser weighting factor
	k_s_	Specular weighting factor
	p	Specular energy
Material/volume	JBIG2-data stream	Lossless compressed binarized voxel data
